# Bacterial Genome Partitioning: N-Terminal Domain of IncC Protein Encoded by Broad-Host-Range Plasmid RK2 Modulates Oligomerisation and DNA Binding

**DOI:** 10.1016/j.jmb.2008.12.016

**Published:** 2009-02-06

**Authors:** Sarah M. Batt, Lewis E.H. Bingle, Tim R. Dafforn, Christopher M. Thomas

**Affiliations:** School of Biosciences, University of Birmingham, Edgbaston, Birmingham B15 2TT, UK

**Keywords:** NTD, N-terminal domain, HTH, helix–turn–helix, AUC, area under the curve, EMSA, electrophoretic mobility shift assay, genome partitioning, plasmid stability, cell cycle, ATPase, protein–protein interaction

## Abstract

ParA Walker ATPases form part of the machinery that promotes better-than-random segregation of bacterial genomes. ParA proteins normally occur in one of two forms, differing by their N-terminal domain (NTD) of approximately 100 aa, which is generally associated with site-specific DNA binding. Unusually, and for as yet unknown reasons, *parA* (*incC*) of IncP-1 plasmids is translated from alternative start codons producing two forms, IncC1 (364 aa) and IncC2 (259 aa), whose ratio varies between hosts. IncC2 could be detected as an oligomeric form containing dimers, tetramers and octamers, but the N-terminal extension present in IncC1 favours nucleotide-stimulated dimerisation as well as high-affinity and ATP-dependent non-specific DNA binding. The IncC1 NTD does not dimerise or bind DNA alone, but it does bind IncC2 in the presence of nucleotides. Mixing IncC1 and IncC2 improved polymerisation and DNA binding. Thus, the NTD may modulate the polymerisation interface, facilitating polymerisation/depolymerisation and DNA binding, to promote the cycle that drives partitioning.

## Introduction

ParA ATPases are a ubiquitous family of proteins associated with directed movement within the bacterial cell of other proteins with or without additional attached macromolecules, such as DNA.^1,2^ They are best known for their role in plasmid and chromosome partitioning, where they work with a ParB protein that binds to a centromere-like sequence within the DNA molecule to be partitioned.^3,4^ They are also associated with directing the segregation of molecular machines, such as chemotaxis protein factories.^5^ Their ability to bind and hydrolyse ATP apparently provides the energy required to drive this movement.^6^ For actin-like ParM ATPases, it is proposed that ParM ATP is added to the outer ends of a ParM filament and that paired ParR–*parC* complexes (the centromere binding protein and DNA, respectively) bind to this and favour ATP hydrolysis to ADP, which loosens the binding, allowing the plasmid–ParR complexes to separate and follow the growing tips of the ParM filament.^7–11^ There is a body of evidence demonstrating that ParB proteins can also pair their binding sites^12^ and that ParA proteins form filaments^13–16^ and show dynamic movement within the bacterial cell.^16^ The recent ParM–ParR–*parC* models referred to above may also apply to these systems, but this is not yet clear.

**Fig. 1:**
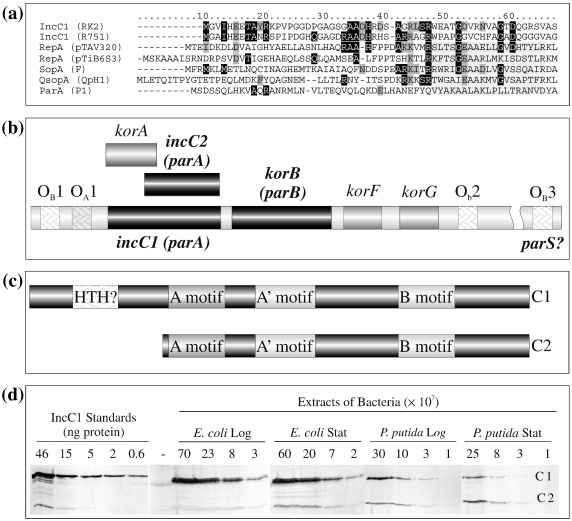
Overview of the IncP-1 system. (a) Alignment of the NTDs of several ParA homologues. The HTH motif is marked by a light gray box. Significant identical or conserved positions are marked with a black or dark gray box. (b) Map of the partitioning operon of RK2 showing the KorB binding sites (OBs) studied previously as possible centromere-like sequences. (c) Cartoon of IncC1 and IncC2 proteins showing the possible HTH and Walker ATPase motifs. (d) Western blotting of extracts of *E. coli* and *P. putida* probed with antibodies to IncC as described in Materials and Methods.

Alignment of the ParA protein sequences identifies two major forms depending on whether the protein starts just before the Walker ATPase box A or includes an N-terminal extension of approximately 100 aa, which generally contains a helix–turn–helix (HTH) motif (Fig. 1a). These longer ParA proteins are plasmid encoded and normally bind DNA specifically to autoregulate their cognate *par* operon—for example, ParA from P1^17^ and SopA from plasmid F.^18^ Specific DNA binding by ParA proteins is stimulated by the presence of ADP and inhibited by ATP.^19^ This ATP–ADP switch in ParA activity is hypothesised to enable ParA to respond to the cell cycle of the host, segregating the plasmid DNA in the presence of ATP, whose levels are higher in exponential phase cells, and repressing expression from the *par* operon once this ATP is hydrolysed to ADP.^20^ This extra domain could therefore be entirely devoted to an autoregulatory function.

In systems with ParA proteins lacking this domain, autoregulation is normally carried out by the cognate ParB protein—for example, those encoded by pTAR^21^ and *Caulobacter crescentus*.^22^ In plasmid RK2, the *par* operon is autoregulated by the combination of the RK2 ParB protein KorB^23^ and KorA, a 101-aa repressor protein encoded by an alternative open-reading frame in a different reading frame within the first section of the RK2 *parA* gene *incC*.^24^ Despite this, the RK2 *parA* gene *incC* does encode a long ParA protein, IncC1, but uniquely, to date, *incC* has a second translational start site, thus producing IncC2, equivalent to short ParA (Fig. 1b and c).^24,25^ IncC1 has not been shown to have DNA binding activity, and sequence alignments in the N-terminal region show only marginal similarity to the HTH region of other ParA proteins (Fig. 1a). Deletion analysis has shown that *incC* functions as a key component of an active partitioning apparatus that is important in *Escherichia coli* as well as other bacteria and that *incC2* is generally sufficient for this activity,^2,26–28^ although this is when the normal control over *incC* and *korB* expression had been removed. In the apparent absence of a role for IncC1 in autoregulation, it is not clear what function encoding both proteins may serve. One approach to increase our understanding of their roles is to determine how the biochemical properties of IncC1 and IncC2 differ. In this study, we compared IncC1 and IncC2, showing that the N-terminal domain (NTD) modulates the polymerisation and non-specific DNA binding properties of this partitioning protein. We suggest that these may be additional functions of NTDs of long ParA proteins that may play an important auxiliary role in the partitioning cycle, possibly by regulating polymerisation and aiding depolymerisation.

## Results

### Production of IncC1 and IncC2 *in vivo*

Although IncC1 and IncC2 are made *in vivo*,^24^ the number and ratio of the two forms have not been estimated quantitatively. Western blotting was therefore used to detect IncC in *E. coli* and *Pseudomonas putida* as examples of distinct hosts (Fig. 1d). Although both forms were detected in each species, the ratios differed significantly: the number of IncC1:IncC2 monomers per log-phase bacterium was 1627:145 for *E. coli* (10:1) and was 496:487 for *P. putida* (1:1). In stationary-phase bacteria, the number of IncC1:IncC2 monomers was 797:219 for *E. coli* (4:1) and was 56:162 for *P. putida* (1:3). While genetic analysis has shown that IncC2 is sufficient for partitioning,^26,28^ these results suggest that IncC1 may actually play the dominant role in some contexts.

### The NTD favours dimer formation by IncC1 in the presence of nucleotides

**Fig. 2:**
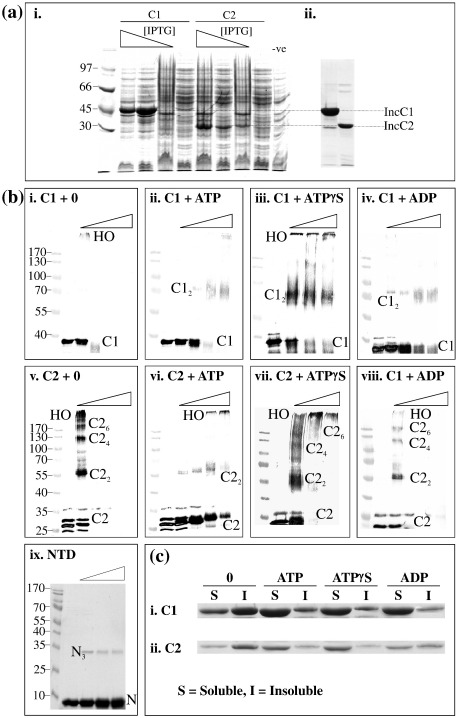
Purification and oligomerisation of IncC1/IncC2. (a) Overexpression and purification of IncC: (i) Comparison of the overexpression of His-IncC1 (C1) and IncC2 (C2) in BL21 and with decreasing IPTG concentrations of 1, 0.5, 0.1 and 0 mM. (ii) Elution samples from nickel-agarose column. (b) Glutaraldehyde cross-linking of IncC1 and IncC2 in the presence of nucleotides. Triangles indicate the increasing percentage of glutaraldehyde added (0, 0.001%, 0.01%, 0.05% and 0.1%; except for ATPγS and NTD, which have no 0.001%). Samples were separated by 9% SDS-PAGE, except for the NTD sample, which was separated by 14% SDS-PAGE. Molecular weight markers are in kilodaltons. HO indicates higher-order structures; C1, IncC1; C2, IncC2; and N, NTD. Subscripts denote the oligomeric state of the proteins. (c) Pelleting studies to show the solubility of IncC1 and IncC2 in the absence (0) or presence of 2 mM nucleotides. S indicates soluble; I, insoluble.

IncC1 and IncC2 proteins were purified to > 90% as described in Materials and Methods (Fig. 2ai and ii). The purified proteins were relatively unstable in the absence of a nucleotide and tended to precipitate when stored for a number of days at 4 °C. The oligomeric state was initially determined using glutaraldehyde cross-linking (Fig. 2b). In the absence of added nucleotides, IncC1 and IncC2 were converted to high-molecular-weight complexes that would not enter the gel (Fig. 2bi and v); however, reproducibly, at low glutaraldehyde concentrations, IncC2 was detected in lower oligomeric forms. In the presence of ATP, IncC1 and IncC2 became more soluble (Fig. 2c) and a dimer was detected as a dominant form but with more IncC2 in monomeric form (Fig. 2bii and vi). With ATPγS and ADP, monomeric and dimeric forms were dominant for IncC1, while only high-molecular-weight forms were detected for IncC2 (Fig. 2biii, iv, vii and viii).

ATPase assays performed as described in Materials and Methods showed that while IncC1 and IncC2 do have significant activity at 37 °C (approximately 30 nM ATP hydrolysed/μM IncC/min), they show no activity at 4 °C, the temperature at which all cross-linking, area-under-the-curve (AUC) and electrophoretic mobility shift assay (EMSA) experiments were carried out. Thus, hydrolysis of ATP during the experiments should not be a complicating factor in the interpretation of the results.

To determine whether the NTD of IncC1 (amino acids 1–105) forms a self-sufficient dimerisation domain, we expressed, purified and glutaraldehyde cross-linked it alone (Fig. 2bix). Much of the protein remained monomeric (11 kDa), while a small amount of the protein formed trimers (33 kDa). By velocity sedimentation analysis, this domain appeared entirely monomeric, independently of whether ATP was present or not (data not shown). Thus, the NTD does not provide an independent dimerisation domain but modulates the activity of residues present in IncC2.

**Fig. 3:**
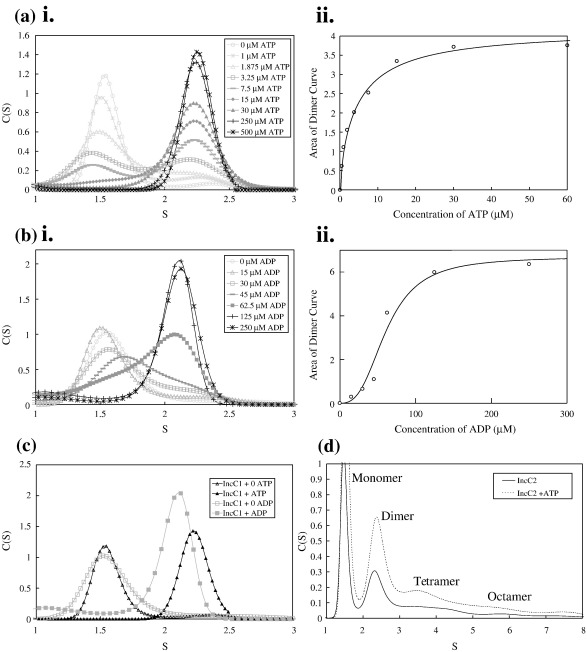
Velocity sedimentation of IncC proteins. (a-i) ATP titrated onto IncC1 (raw analysis). (a-ii) Graph of the area under the IncC1 dimer curve plotted against ATP concentration (micromolar). (b-i) ADP titrated onto IncC1 (raw analysis). (b-ii) Graph of the area under the IncC1 dimer curve plotted against ADP concentration (micromolar). (c) Comparison of the *S* values for the IncC1-ATP dimer and the IncC1-ADP dimer. (d) IncC2 in the presence and that in the absence of 2 mM ATP.

Velocity sedimentation experiments with 30 μM IncC1 and varying ATP concentrations confirmed that IncC1 forms a dimer (mass = 80 kDa, 2.3*S*) in the presence of this nucleotide; in the absence of ATP, the soluble IncC1 that is detectable is exclusively monomeric (mass = 40 kDa, 1.6*S*) (Fig. 3ai). Plotting the dimer concentration at increasing ATP concentrations (Fig. 3aii) enabled the apparent dissociation constant (*K*_app_) of IncC1 binding ATP to be calculated as 7.7 μM, considerably lower than the apparent *K*_d_ values for other ParA proteins, which are in the range of 33–100 μM.^13,14,19,22^ Magnesium was important for coordinating the nucleotide, with the *K*_app_ being 130-fold higher (940 μM for 20 μM IncC1) when Mg^2+^ was absent (data not shown). With magnesium, all of IncC1 is dimeric at or above 30 μM ATP, approximately one ATP molecule per IncC1 monomer. ADP also caused the formation of IncC1 dimers (Fig. 3bi), but with a higher *K*_app_ (Fig. 3bii; 54 μM). Hill analysis^29^ of the data indicated that there was no cooperativity with ATP, whereas the ADP-induced dimerisation was highly cooperative involving two sites, the second of which was calculated to have a 1000-fold higher affinity.

The IncC1 dimer formed in the presence of ADP has a sedimentation value different from that formed with ATP (2.1 ± 0.02*S* compared with 2.3 ± 0.08*S*; Fig. 3c), which could imply a different conformation. By contrast, in similar experiments, IncC2 showed the presence of low levels of dimers, tetramers and octamers, independently of the presence of nucleotides (Fig. 3d), although the dimeric form was increased by the presence of ATP. Deletion of even 23 N-terminal amino acids resulted in properties similar to IncC2 (data not shown). Thus, an intact NTD is essential for the very clear differences in oligomerisation between IncC1 and IncC2: it appears to prevent oligomerisation in the absence of nucleotide and limits it to dimerisation in the presence of ATP.

### IncC1 exhibits higher ATP-stimulated affinity for DNA than IncC2

**Fig. 4:**
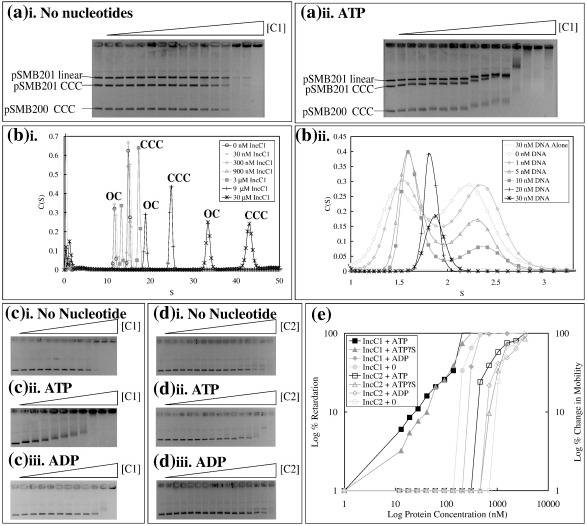
DNA binding activity of IncC1. (a) Competition band shifts of IncC1 with linear and supercoiled DNAs plus and minus the central control region from RK2 (pSMB201, plasmid with central control region from RK2; pSMB200, pSMB201 without the central control region). Triangles represent increasing concentrations of each IncC1 in the retardation (in 1.5-fold steps, beginning with 18 nM). Band shift assays performed with supercoiled and digested pSMB200, same as pSMB201 but without the central control region and supercoiled pSMB200 on agarose gel (0.7%). (b-i) Velocity sedimentation of IncC1 titrated onto DNA. A 15 nM mixture of supercoiled (CCC) and open circular (OC) pSMB201 DNAs was titrated with IncC1. (ii) Velocity sedimentation of DNA titrated onto IncC1. IncC1 (34 μM) was titrated with supercoiled and open circular pSMB201 DNAs. A concentration of ATP was added such that there was a 1:1 ratio of IncC1 monomer/dimer. (c) Band shifts of IncC1 with no nucleotide, ATP and ADP. Band shift assays performed with supercoiled pSMB201 DNA on agarose gel (0.7%) with increasing concentrations of IncC1 (in 1.5-fold steps, beginning with 12 nM). (d) Band shifts of IncC2 with no nucleotide, ATP and ADP. Band shift assays performed with supercoiled pSMB201 DNA on agarose gel (0.7%) with increasing concentrations of IncC2 (in 1.5-fold steps, beginning with 12 nM). (e) Graphical representation of band shifts of IncC1 and IncC2 with various nucleotides. For band shifts with ATP and ATPγS, the percentage of change in mobility was calculated as the percentage of reduction in the distance of the DNA band from the well. For band shifts with no nucleotide and ADP, the percentage of retardation was calculated by the reduction of the DNA band's intensity, which was analysed using Quantity One (BioRad).

Although no DNA binding by IncC was observed previously,^30^ we reinvestigated this using a mixture of whole plasmids with and without the central control region of RK2, which was thought most likely to contain binding sites. In the absence of ATP, IncC1 (Fig. 4ai) and IncC2 (data not shown) were found to favour binding to supercoiled DNA, which was fully retarded in EMSAs at twofold lower protein concentrations than the linear DNA. No preference for plasmids containing RK2 central control region DNA with pSMB200 *versus* pSMB201 was observed. Velocity sedimentation performed with a 15 nM mixture of supercoiled and open circular pSMB201 DNAs (Fig. 4bi) also indicated an IncC1 preference for supercoiled DNA: the sedimentation coefficient of the peak corresponding to supercoiled DNA was more sensitive to increasing IncC1 concentrations than that of the open circular form. A change in sedimentation rate was not observed until the concentration of IncC1 reached between 0.9 and 3 μM, consistent with the concentration at which EMSA showed the transition from unretarded state to complete retardation (Fig. 4ai).

By contrast, the presence of ATP caused gradual retardation in EMSA experiments with both linear and supercoiled plasmid DNAs from as low as 10 nM IncC1 (Fig. 4aii). Velocity sedimentation of increasing concentrations of supercoiled DNA in the presence of ATP, to create an equal mixture of IncC1 monomers and dimers (Fig. 4bii), showed that it is the IncC1-ATP dimer that binds to DNA, while the concentration of the monomer peak remains constant. All of the dimer bound to the DNA at a concentration of 10–20 nM DNA, corresponding to one dimer of IncC1 per 10–20 bases of DNA. Once all of the dimer was bound (when there was excess of double-stranded DNA), the *S* value of the remaining IncC1 was seen to shift from 1.6 to 1.9, midway between the *S* values of the monomer and the dimer, suggesting a fast equilibrium between monomer and dimer. Since a mixture of IncC1 and ATP alone does not give such fast exchange between dimer and monomer, this implies that the presence of uncoated DNA may promote dimer/monomer exchange.

ATP increases IncC1 affinity for DNA by more than 30-fold (Fig. 4c) and alters the mode of DNA binding: in the absence of ATP, the DNA appears to be shifted straight to the top of the gel, whereas in the presence of ATP, DNA mobility decreases slowly, implying progressive accumulation of protein on the plasmid DNA. ATPγS also increased IncC1's affinity for DNA by more than 15-fold. The mode of DNA binding in the presence of ADP is similar to that in the absence of any nucleotide, and it has a slight inhibitory effect of approximately 1.5-fold on DNA binding, which is consistent with ADP-IncC1 having a conformation different from that of ATP–IncC1 (Fig. 4c). A semi-logarithmic graph of the data demonstrated that in the presence of ATP, IncC1 binding to DNA is biphasic, with the transition between the two phases occurring at a concentration sufficient for one IncC1 dimer every 20 bp (approximate). This is also approximately the concentration at which the lower-affinity forms of IncC1 cause retardation. Hill analysis^29^ of the data indicated that, with both ATP and ATPγS, there is cooperativity involving only two sites, presumably those present in the IncC1 dimer. For free IncC1 or in the presence of ADP, there is very strong cooperativity involving multiple sites, with as many as 18 predicted from one data set. In the presence of ATP, mobility of the entire DNA gradually changed in a concerted way, suggesting negative cooperativity such that distribution of IncC1 evenly between the DNA molecules is favoured, perhaps due to conformational changes in the DNA as a result of IncC1 binding. Alternatively, this could be due to a fast equilibrium of IncC1 continually binding to the DNA and then detaching.

ATP also stimulated IncC2 binding to DNA, but the stimulation was only 2-fold and IncC2 also had a much lower overall affinity for DNA than IncC1 (Fig. 4d). In the absence of nucleotides, the difference between the affinities of the two proteins is approximately 7-fold: 0.5 μM for complete retardation by IncC1 compared with 3.5 μM for IncC2. In the presence of ATP, the difference between the affinities of the two proteins for DNA is 18-fold. Thus, the NTD of IncC1 has a major effect on IncC DNA binding properties. Interestingly, ATPase activity of both IncC1 and IncC2 was stimulated (about 45% from about 30–42 nM ATP hydrolysed/μM IncC/min) by the presence of either CCC or OC DNA, although not so much as when KorB was also present and the DNA was a plasmid containing an O_B_ site (more than 2-fold; unpublished data). It therefore seems unlikely that interaction of IncC1 or IncC2 with DNA is likely to switch on its ATPase activity in a major way.

### Analysis of IncC1 NTD

**Fig. 5:**
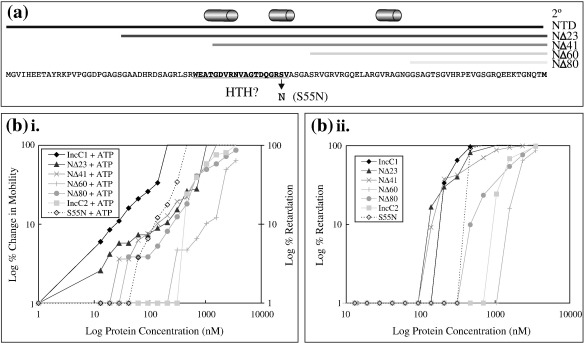
DNA binding by N-terminal deletion mutants of IncC1. (a) Diagram of IncC1 N-terminal deletion mutants. All delta (Δ) N-terminal mutants continue to the end of IncC1 (not shown in diagram). Amino acids corresponding to the HTH domain on other ParA proteins are in boldface. The predicted secondary structure (labelled 2°) is shown above (cylinders correspond to α-helices).^33^ (b) Graphs of band shifts performed with the IncC1 N-terminal deletion mutants using supercoiled pSMB201 DNA. For band shifts with ATP, the percentage of change in mobility was calculated as the percentage of reduction in the distance of the DNA band from the well. For band shifts with no nucleotide (as well as IncC2 and NΔ60 with ATP), the percentage of retardation was calculated by the reduction of the DNA band's intensity, which was analysed using Quantity One (BioRad).

Deletion mutants (Fig. 5a) were constructed in approximately 20-aa steps, with endpoints defined by the predicted secondary structures of the NTDs of other ParA proteins, ensuring that complete α-helices and β-sheets were included or deleted from each mutant derivative. The predicted structure of the IncC1-NTD was also consistent with the known structure of the Ffh signal recognition particle protein, which shows sequence similarities to ParA proteins.^31,32^ EMSAs with supercoiled plasmid DNA (Fig. 5b) showed that even removal of 23 aa from the N-terminus decreased efficiency of DNA binding: by 1.5-fold in the absence of any nucleotide and by 5-fold in the presence of ATP. Deletion of 41 aa had a slight additional effect (2-fold and 8-fold in the absence and in the presence of ATP, respectively), but the greatest decline in DNA binding was shown by IncC1-NΔ60 and IncC1-NΔ80, both of which no longer contain the region that in other ParA proteins encodes the putative HTH domain. These mutants both retarded the DNA at similar concentrations as with IncC2—a difference of 7-fold in the absence of any nucleotide and that of 18-fold in the presence of ATP as compared with wild-type IncC1. There was also a difference in the gradual reduction in DNA mobility in the presence of ATP: IncC1-NΔ23, IncC1-NΔ41 and IncC1-NΔ80 all behaved similarly to IncC1, while IncC1-NΔ60 behaved more like IncC2, retarding the DNA over a narrower protein concentration, which demonstrates that binding is more cooperative. A point mutation in the NTD of IncC1 (S55N) did not alter the basic affinity of IncC1 for supercoiled DNA but did slightly reduce the ability of ATP to stimulate binding (Fig. 5b). Tests for the dimerisation of the deletion derivatives revealed that even the smallest deletion (23 aa) resulted in complete loss of ATP-stimulated dimerisation possessed by IncC1 (data not shown). When the NTD of IncC1 was purified alone, it did not retard the DNA at all at any of the concentrations tested (data not shown). Thus, the influence of the NTD on IncC behaviour depends on the whole domain being intact and in the context of a complete IncC protein.

**Fig. 6:**
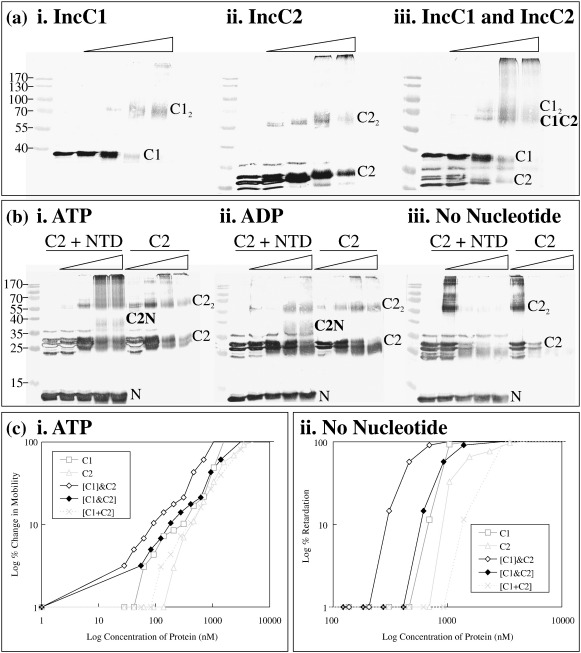
Interactions between IncC1 and IncC2. (a) Western blot analysis of glutaraldehyde cross-linked complexes between IncC1 and IncC2 in the presence of ATP. (b) Western blot analysis of glutaraldehyde cross-linked complexes between IncC2 and the NTD of IncC1 in the presence of various nucleotides. Blots were probed with anti-IncC. Triangles indicate the increasing percentage of glutaraldehyde added (0%, 0.001%, 0.01%, 0.05% and 0.1%). Samples were separated by 9% (for IncC1 gels) and 14% (for NTD gels) SDS-PAGE. Molecular weight markers are in kilodaltons. HO indicates higher-order structures; C1, IncC1; C2, IncC2; and N, IncC1 NTD. Superscripts denote the oligomeric state of the proteins. (c) Graph of band shifts performed with IncC1, IncC2 and a mixture of IncC1 and IncC2. For band shifts with ATP, the percentage of change in mobility was calculated as the percentage of reduction in the distance of the DNA band from the well. For band shifts with ADP, the percentage of retardation was calculated by the reduction of the DNA band's intensity, which was analysed using Quantity One (BioRad). [C1]&C2 indicates both proteins in the same band shift using the concentration of IncC1; [C1&C2], both proteins in the same band shift using the concentration of both proteins; and [C1 + C2], separate band shift data from IncC1 and IncC2 added together using both proteins' concentrations.

To determine whether the NTD of IncC1 interacts with IncC2, we cross-linked them in the presence of various nucleotides (Fig. 6b). In the presence of either ATP or ADP, the NTD was found to interact with IncC2, forming a new species with an estimated relative molecular mass of 41 kDa, which was not seen in the absence of nucleotides. This correlates with the predicted role of the NTD in regulating ATP/ADP-stimulated dimerisation in IncC1, suggesting that it interacts with an area of IncC2 that is exposed when the protein binds a nucleotide.

### IncC2 enhances DNA binding by IncC1

IncC1 and IncC2 were mixed and glutaraldehyde cross-linked in the presence of various nucleotides (Fig. 6a). Western blots showed that the two proteins form a complex corresponding to a heterodimer, with an estimated relative molecular mass of 68 kDa. IncC1 homodimers (78 kDa) were also visible. This species could also be seen in the absence of nucleotide, albeit only at the lowest glutaraldehyde concentration (0.001%) as part of a ladder of IncC2 oligomers, suggesting that it may originate from the insoluble or high-molecular-weight material than a real heterodimer in this case. Interestingly, with ATP, the presence of IncC1 reduced the concentration of monomeric IncC2 at higher glutaraldehyde concentrations, and there were also additional high-molecular-weight complexes visible, as compared with IncC2 alone. These suggest that IncC1 stimulates IncC2 polymerisation in the presence of ATP. In the presence of ATP, the IncC1 dimer and heterodimer with IncC2 were less prevalent at higher glutaraldehyde concentrations, suggesting that they were incorporated into growing polymers. This does not occur to the same extent with ADP (data not shown), implying that ATP is the nucleotide required for polymerisation.

With supercoiled pSMB201, DNA retardation by a 1:1 mixture of IncC1/IncC2 as well as each protein demonstrated that IncC2 enhances the DNA binding of IncC1, independently of the nucleotide present (Fig. 6c). Thus, the IncC1/IncC2 mixture has a higher affinity for the DNA than IncC1 alone, and this is not dependent on the form of nucleotide bound. In the presence of ATP, for example, 54 nM concentration of the IncC1/IncC2 mixture begins to retard the DNA, whereas 62 nM IncC1 alone was required to retard the DNA to the same extent. In the presence of ADP, the IncC1/IncC2 mixture showed approximately a 1.6-fold higher affinity than IncC1.

## Discussion

Biochemical studies on a variety of ParA proteins have revealed not only many similarities but also distinct differences in protein properties, some of which may be due to the approximately 100-aa NTD that is present in some but not all ParA proteins. Comparative studies on the naturally occurring RK2 IncC1 and IncC2 proteins reported here show that the NTD of IncC1 can profoundly influence the activity of IncC2, the shorter protein, despite the latter apparently being functional for active partitioning *in vivo*.^26^ A major difference of IncC from a number of other ParA proteins is that the addition of ATP aids the solubility of the IncC proteins, apparently priming them to enter the partitioning cycle by addition to the growing ParA filament, whereas for many other ParA proteins, ATP promotes polymerisation independently of other key parts of the partitioning machinery. Thus, IncC may represent a currently unique biochemical model of a partitioning cycle based on polymerisation of soluble ParA initiated by the cognate ParB bound to its centromere-like DNA sequence. These differences may reflect both the assays performed and how polymerisation is measured, but since our methods were based on previous reports with a variety of systems, it may be that they represent inherent properties of the RK2 system and lay the basis for investigating the depairing and separation stages of the partitioning cycle.

**Fig. 7:**
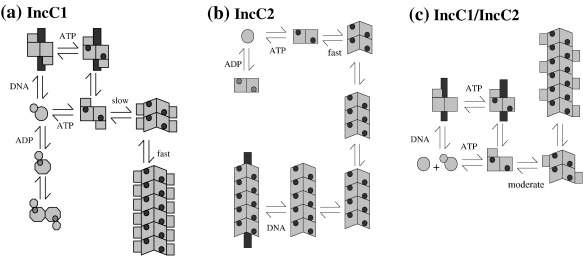
Summary of polymerisation and DNA (dark vertical bar) binding activities observed for IncC1 (a), IncC2 (b) and the IncC1/IncC2 mixture (c) in the presence of nucleotide (small darker circles) ATP or ADP (for IncC1). The large light square/circle represents the IncC2 segment, while in (a) and (c), the small light square/circle represents the NTD. A key element of the model is that while ATP binding causes a conformational change for each protein, the change with ATP configures a DNA binding site, whereas that with ADP does not. Also note that the NTD is clamped onto the IncC2 portion by ATP but must move or be moved out of the way to allow further polymerisation.

Another feature of our data is that the two *incC* products show such distinct oligomerisation and DNA binding properties (summarised in Fig. 7). ATP and ADP promote IncC1 to form dimers, albeit with distinct properties—only ATP binding gives high, non-sequence-specific affinity for DNA. Thus, apart from the lack of ADP-dependent sequence-specific DNA binding (which correlates with IncC1 NTD lacking strong similarity to the HTH DNA recognition motif; Fig. 1a), IncC1 has many properties typical of other long ParA proteins.^3,14,17,19^ By contrast, IncC2 lacks the high-affinity non-specific DNA binding activity typical of short ParA proteins. It forms dimers less readily than IncC1 while having a greater tendency to continue polymerising once dimers are formed as indicated by both cross-linking and AUC. Since IncC2 is sufficient for partitioning,^26^ the strong non-sequence-specific DNA binding cannot be essential for the partition cycle but may increase efficiency in some way—for example, by increasing local concentrations of ParA or reducing ATP hydrolysis in the absence of the cognate ParB. Understanding how the NTD of IncC1 modulates ParA protein activity may establish how the equivalent domain in other ParA proteins contributes to the partitioning cycle.

The entire NTD is necessary for IncC1's greater tendency (compared with IncC2) to form dimers irrespective of the bound nucleotide, but the 105-aa region does not dimerise autonomously. Therefore, it seems unlikely that these proteins arose from the fusion of a small dimeric DNA binding protein with a standard short ParA (IncC2) protein. Our data also suggest that the IncC1 NTD forms a separate domain that, in the presence of a nucleotide, changes position or conformation and associates more closely with the common domain of IncC1 and IncC2. This appears to potentiate the dimerisation interface but also blocks or alters surfaces needed for further polymerisation since IncC2, lacking this domain, has a greater tendency to oligomerise to high-molecular-weight forms. The importance of the nucleotide in determining active conformation is emphasised by the differences in sedimentation of IncC1-ATP and IncC1-ADP, as well as their DNA binding properties. Mapping the surfaces needed for the macromolecular interactions that IncC may be involved in (IncC–IncC, IncC_2_–IncC_2_, IncC–DNA, IncC–KorB and IncC2–NTD) should help make more sense of the oligomerisation process and its control. However, the current state of knowledge is consistent with the idea that IncC-ATP is recruited to the filament that drives partitioning but then undergoes ATP hydrolysis and conformational change, which is a key step in the cycle proposed recently.^9,11^

Although it is conceivable that the alternative *incC* translation starts could simply be a flexible way to supply sufficient IncC in different species, Western blotting showed that IncC1 can be either dominant (IncC1/IncC2 in *E. coli* is 10:1) or equal to IncC2 (*P. putida*) depending on the host. This implies that the distinct properties of IncC1, particularly the non-specific high affinity for DNA, are important and suggests that IncC1 and IncC2 are not totally interchangeable. While the above ratios suggest that IncC1 might be the “senior partner” in the system, the data also suggest that IncC1/IncC2 heterodimers bind DNA as efficiently as or better than IncC1 alone; hence, producing a mixture may be the real purpose of the system. The role of non-sequence-specific DNA binding has been recently discussed,^14^ but we must repeat some of the points here. First, although ATP-stimulated non-specific DNA binding for ParA protein from *Bacillus subtilis* (Soj) is important for polymerisation to form filaments,^32^ our data show that this is not the case for IncC. Second, as for the SopA protein of F,^14^ non-specific DNA binding may reduce polymerisation. DNA binding of IncC1-ATP certainly appears to be non-cooperative and DNA-bound IncC could thus act as a store of the protein until it is required during segregation. KorB bound to DNA in a particular way that is characteristic of paired plasmid molecules may release IncC from DNA and stimulate polymerisation. A third possibility is that IncC may condense DNA, compacting the multiple *parS* sites to form an organised nucleoprotein complex, a role predicted for Soj in *B. subtilis*.^34^ Chromosome condensation is important during eukaryotic mitosis, so it would not be surprising for the same to be true for prokaryotes. Compaction could prevent sister origin regions from becoming intertwined and decrease the resistance as they are moved through the cytoplasm.^34^ A fourth possibility is that DNA forms a scaffold on which the partitioning apparatus assembles. Future studies looking into the effect on partitioning of point mutations that inactivate this property in IncC1 but leave other properties intact should be instructive in distinguishing between these possibilities.

The key similarities and differences between IncC1 and IncC2 reported in this article are summarised in Fig. 7. Despite their differences, IncC1 and IncC2 interact, producing heterodimers, regardless of their nucleotide-bound state. Band shift experiments demonstrated that the DNA binding ability of the IncC1/IncC2 mixture, possibly the heterodimer itself, was greater than that of IncC1 or IncC2 homodimers. The ADP binding data suggest that conformational change in one subunit of a dimer can be transmitted to the other subunit. Therefore, the conformation promoted by an NTD on only one subunit of a dimer may increase DNA binding strength more than an NTD on both subunits. Alternatively, an IncC1 dimer and an IncC2 dimer together may bind more strongly than a homogeneous mixture of homodimers. This may increase the effectiveness of the relatively low levels of IncC in the bacterial cell. Interaction between the IncC proteins may promote polymerisation of IncC2, either through nucleation by IncC1 or due to the formation of the heterodimer. Our data with IncC2 suggest that polymerisation occurs through dimer intermediates. The observation that nucleotide binding promotes binding of the non-covalently attached NTD to IncC2 suggests that these domain–domain interactions are responsible for both the potentiation and the inhibition of different properties. It is plausible that the interaction between the two forms is particularly potent because the heterodimer may gain from the positive contribution of the NTD on one subunit to DNA binding without being dominated by the negative influence of the NTD on polymerisation, which would occur if present on both subunits. Seeing the NTD as a critical regulator of this process may help in understanding features of the different systems and direct further experiments to establish the interactions that control the various stages of the partitioning cycle.

## Materials and Methods

### Bacterial strains and growth conditions

*E. coli* strains used in this study were DH5α (*supE44* Δ*lacU61 [ϕ80lacZ* Δ*M15] hsdR17 recA1 endA1 gyrA96 thi-1 relA1*) for constructing and propagating plasmids^35^ and BL21 (F^−^ *ompT*, *hsdS_B_ [r_B_^−^m_B_^−^] gal*, *dcm* [phage DE3]) for overexpression of proteins from expression vectors with the bacteriophage T7 promoter (Novagen). The bacteria were grown in Luria broth (LB) or on LB agar.^36^ Growth medium was supplemented with antibiotics where appropriate. Incubation was carried out at 37 °C unless otherwise stated. Plasmids used or created during the course of this work are listed in Table 1.

### Protein purification

His_6_-IncC1, His_6_-IncC2 and N-terminal deletion derivatives were overexpressed in C600 BL21 from a modified pET28a vector.^30^ Four hundred-milliliter cultures were grown to an OD_600_ of ∼ 0.4–0.6 and induced with 1 mM IPTG for 4 h, prior to harvesting the cells at 18,600***g***. All incubation procedures were carried out at 25 °C to avoid insolubility observed at 37 °C. Purification was carried out under native conditions using a nickel-agarose column (QIAGEN) according to the manufacturer's instructions. Glycerol (10%) was added to the wash and elution buffers to increase protein solubility. Purity was judged by SDS-PAGE.^37,38^ Thrombin cleavage to remove the His tag was carried out using a Thrombin Cleavage Capture Kit (Novagen).

### Assessment of IncC solubility

Either IncC1 or IncC2 (25 μg) was diluted to a total volume of 15 μl with 10× buffers, producing a final concentration of 10 mM MgCl_2_ and 25 mM sodium phosphate, pH 7.5. In some experiments, the buffer also contained nucleotides as necessary (2 mM concentration of ATP, ADP or ATPγS). Samples were incubated for 30 min at 37 °C and then centrifuged at 14,000*g* for 30 min to pellet any precipitated protein. The pellets were resuspended in loading buffer, and 5 μl of loading buffer was added to the supernatant. Samples were analysed by 12% SDS-PAGE.

### Glutaraldehyde cross-linking

Each protein (5 μg) was diluted to a total volume of 50 μl with 10× buffers, 100 mM MgCl_2_ and 0.25 M phosphate buffer, pH 7.5, and H_2_O, producing a final concentration of 10 mM MgCl_2_ and 25 mM phosphate buffer, pH 7.5, with and without 2 mM ATP, ADP or ATPγS. After 30 min incubation at 37 °C, glutaraldehyde was added to concentrations of 0%, 0.01%, 0.05% and 0.1% before 30 min further incubation at 30 °C. Cross-linking was quenched with 0.14 M ethanolamine, pH 8.0, before analysis by SDS-PAGE. Gels were Western blotted as described below.

### Western blotting

Proteins were first separated by SDS-PAGE and then blotted onto a nitrocellulose membrane (Hybond-C pure) using Mini Trans-Blot^®^ Electrophoretic Transfer Cell (BioRad) and incubated with antibody using standard methods.^35^ To visualise antibody binding, we used an Amplified Alkaline Phosphatase Goat Anti-Rabbit Immunoblot Assay kit (BioRad) according to the manufacturer's instructions.

### Purification of antibodies

Antibodies cross-reacting with other *E. coli* and *P. putida* proteins were first removed as described previously.^39^ The antibodies were concentrated and further purified by affinity for the specified protein as described previously.^27^

### Large-scale isolation of plasmid DNA

DNA was isolated based on large-scale alkaline lysis,^40^ followed by caesium chloride/ethidium bromide density gradient centrifugation,^35^ which separates the plasmid DNA from the chromosomal DNA, after which the ethidium bromide is removed.

### Analytical ultracentrifugation

Sedimentation velocity experiments were carried out in a Beckman XL-A analytical ultracentrifuge equipped with absorbance optics. Protein samples were dialysed overnight into buffer (50 mM sodium phosphate, pH 8.0, and 100 mM NaCl), and then any precipitated protein was cleared by centrifugation at 14,000*g* for 30 min. Samples (with a protein concentration of approximately 0.1 mg/ml) were loaded into one channel of cells with two-channel Epon centrepieces and quartz windows. The reference (buffer alone) was loaded into the other channel. Samples were centrifuged at 40,000 rpm, 4 °C, using an An50Ti rotor. Scans of an absorbance wavelength of 280 nm were taken every 6 min. Partial specific volumes were calculated using the program SEDNTERP.^41^ The data were analysed using the program SEDFIT.^42^ Sedimentation coefficient distributions were calculated using Lamm equation modelling implementing maximum entropy regularisation. A total of 100 scans for each sample was analysed, which represents the full extent of sedimentation of the sample. When a mixture of linear DNA *versus* supercoiled DNA was analysed, the latter sedimented faster, and IncC titrated in the relative positions of the peaks allowed clear identification of the two forms.

### Electrophoretic mobility shift assays

DNA (0.4 nM, supercoiled or digested) was incubated with varying protein concentrations with or without nucleotides (2 mM concentration of ATP, ADP ATPγS or GTP). Proteins and DNA were diluted to a total volume of 20 μl with 10× buffers, producing a final concentration of 10 mM MgCl_2_, 25 mM phosphate buffer, pH 7.5, and 0.2 mg/ml of bovine serum albumin. Samples were incubated for 30 min at 37 °C and then separated on an agarose gel at 4 °C, 70 V. Ethidium bromide was not added to the agarose as it intercalates with DNA and could alter protein binding; consequently, gels were post-stained for 30 min in an opaque box containing TAE with ethidium bromide on a tilting table. This was repeated twice for each condition.

### ATPase assays

The ATPase activity of both IncC1 and IncC2 was determined using the detection of released inorganic phosphate after the addition of malachite green reagent.^43^ This allows for the spectrophometric detection of colour change (yellow to green) in the presence of inorganic phosphate. Malachite green reagent [1:1:2:2 ratio of 5.72% (w/v) ammonium molybdate in 6N HCL, 2.32% (w/v) polyvinyl alcohol, 0.08712% (w/v) malachite green and distilled water] was made and left to turn paler before use. Reactions were performed in a total volume of 400 μl in 50 mM Tris–HCl, pH 8.0, 150 mM NaCl, 5% (v/v) glycerol, 1 mM ATP and 10 mM MgCl_2_. Reactions contained varying combinations of 36 μM concentration of IncC1/IncC2, 5 μM concentration of KorB and 75 nM concentration of plasmid pSMB201. Samples were incubated at 37 °C, and 50-μl aliquots were taken every 30 min and terminated by the addition of 200 μl of the malachite green reagent. After an incubation period of 5 min at room temperature, the absorbance of the samples was measured at 630 nm. A calibration curve of 0–200 μM inorganic phosphate standards was constructed, and samples were normalised for acid hydrolysis of ATP caused by the malachite green reagent.

## Figures and Tables

**Table 1:** Plasmids used or constructed in this study

Plasmid	Selectable marker	Size (kb)	Replicon	Properties	Reference
pGBT340	Km^R^	5.4	pMB1	pET-28a with T7 tag removed	Ref. 30
pGBT342	Km^R^	6.4	pMB1	pGBT340 with *incC1* inserted in *MCS* by EcoRI–SalI; The ATG start codon for *korA* has been changed to ACG without altering the amino acid sequence for *incC1*	Ref. 30
pGBT343	Km^R^	6.2	pMB1	pGBT340 with *incC2* inserted in *MCS* by EcoRI–SalI	Ref. 30
pSMB320	Km^R^	6.5	pMB1	pGBT340 with *korB* inserted in *MCS* by EcoRI–SalI	This study
pSMB311	Km^R^	6.4	pMB1	pGBT340 with *incC1*Δ*23* inserted by EcoRI–SalI (23 aa removed from N-terminus)	This study
pSMB312	Km^R^	6.4	pMB1	pGBT340 with *incC1*Δ*41* inserted by EcoRI–SalI (41 aa removed from N-terminus)	This study
pSMB313	Km^R^	6.4	pMB1	pGBT340 with *incC1*Δ*60* inserted by EcoRI–SalI (60 aa removed from N-terminus)	This study
pSMB314	Km^R^	6.4	pMB1	pGBT340 with *incC1*Δ*80* inserted by EcoRI–SalI (80 aa removed from N-terminus)	This study
pSMB315	Km^R^	5.7	pMB1	pGBT340 with *incC1-NTD* inserted by EcoRI–SalI (NTD is N-terminal domain)	This study
pSMB316	Km^R^	6.4	pMB1	pGBT340 with *incC1ts3* inserted by EcoRI–SalI (55S → N 164G → A mutant)	This study
pSMB200	Ap^R^Tc^R^	4.3	pMB1	pBR322 with an oligo inserted into Tc^R^	This study
pSMB201	Ap^R^	10	pMB1	pBR322 with central control region inserted into Tc^R^	This study
